# Insights into Tetrazine–Benzene Cycloadditions

**DOI:** 10.1021/acs.jpca.6c01346

**Published:** 2026-06-03

**Authors:** Tori Demuth, Dennis Svatunek

**Affiliations:** Institute of Applied Synthetic Chemistry, 27259 TU Wien, Getreidemarkt 9, 1060 Vienna, Austria

## Abstract

Arene–tetrazine cycloadditions involving benzene as the dienophile represent an exceptionally rare class of Diels–Alder reactions. In this study, we computationally investigate the origin of the intrinsic reactivity of simple aromatic systems toward tetrazines and identify the factors that enable successful cycloaddition. Our results show that only highly activated tetrazines can overcome the unfavorable orbital overlap associated with the compact π-system of benzene. Energy decomposition analysis reveals that reactivity is governed by a combination of frontier molecular orbital interactions and charge-control effects. Substituent effects strongly influence the activation barrier, particularly when they promote a shift toward a more asynchronous and polar transition state. These findings provide mechanistic insight into a rare mode of arene reactivity and clarify the electronic requirements for enabling tetrazine Diels–Alder reactions with aromatic dienophiles.

## Graphical abstract

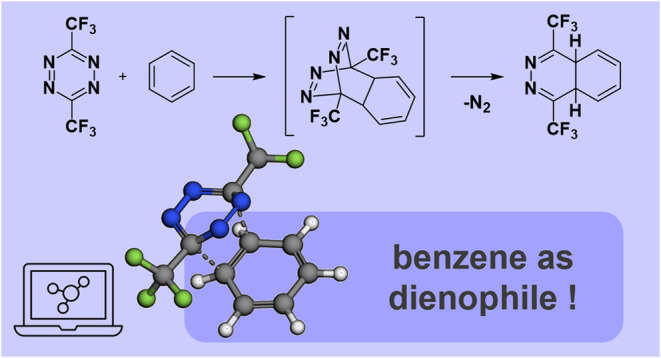

## Introduction

The Diels–Alder reaction is among the most versatile transformations in organic chemistry, enabling the rapid and selective assembly of complex cyclic and heterocyclic frameworks through the formation of two new σ-bonds.^1^ A broad variety of dienes and dienophiles is known, encompassing acyclic and cyclic systems as well as heteroatom-substituted and electronically activated partners. Notably, even weakly aromatic compounds such as furan can participate, highlighting the exceptional scope and adaptability of this transformation.

In contrast, strongly aromatic systems such as benzene and its derivatives generally do not participate in Diels–Alder reactions, owing to the substantial energetic penalty associated with loss of aromaticity. Only a handful of examples have been reported in which highly activated dienophiles engage aromatic dienes. Notable examples include reactions with highly electron-poor fluorinated alkenes^2^ and alkynes,^3–5^ introducing significant geometric strain or enforced proximity,^6–8^ or activation using metal coordination.^9^

Additionally, there are a few reported cases of a benzene derivative acting as a dienophile in a Diels–Alder reaction. Korte and co-workers demonstrated that benzene can react with hexachlorocyclopentadiene under high pressure (1000 bar) and high temperatures (220 to 240 °C).^10^ Inagaki et al. introduced a highly reactive “super-diene” that undergoes cycloaddition with benzene at moderate temperatures (120 °C), followed by fragmentation of the scaffold.^11^ Additionally, some 1,2,4,5-tetrazines are also sufficiently powerful dienes to engage benzene directly. In a landmark 1987 study, Seitz, Hoferichter, and Mohr demonstrated that benzene and substituted benzenes can act as dienophiles in Diels–Alder reactions with the exceptionally electron-poor 3,6-bis­(trifluoromethyl)-1,2,4,5-tetrazine under thermal conditions (Figure 1).^12^

**Figure 1:**
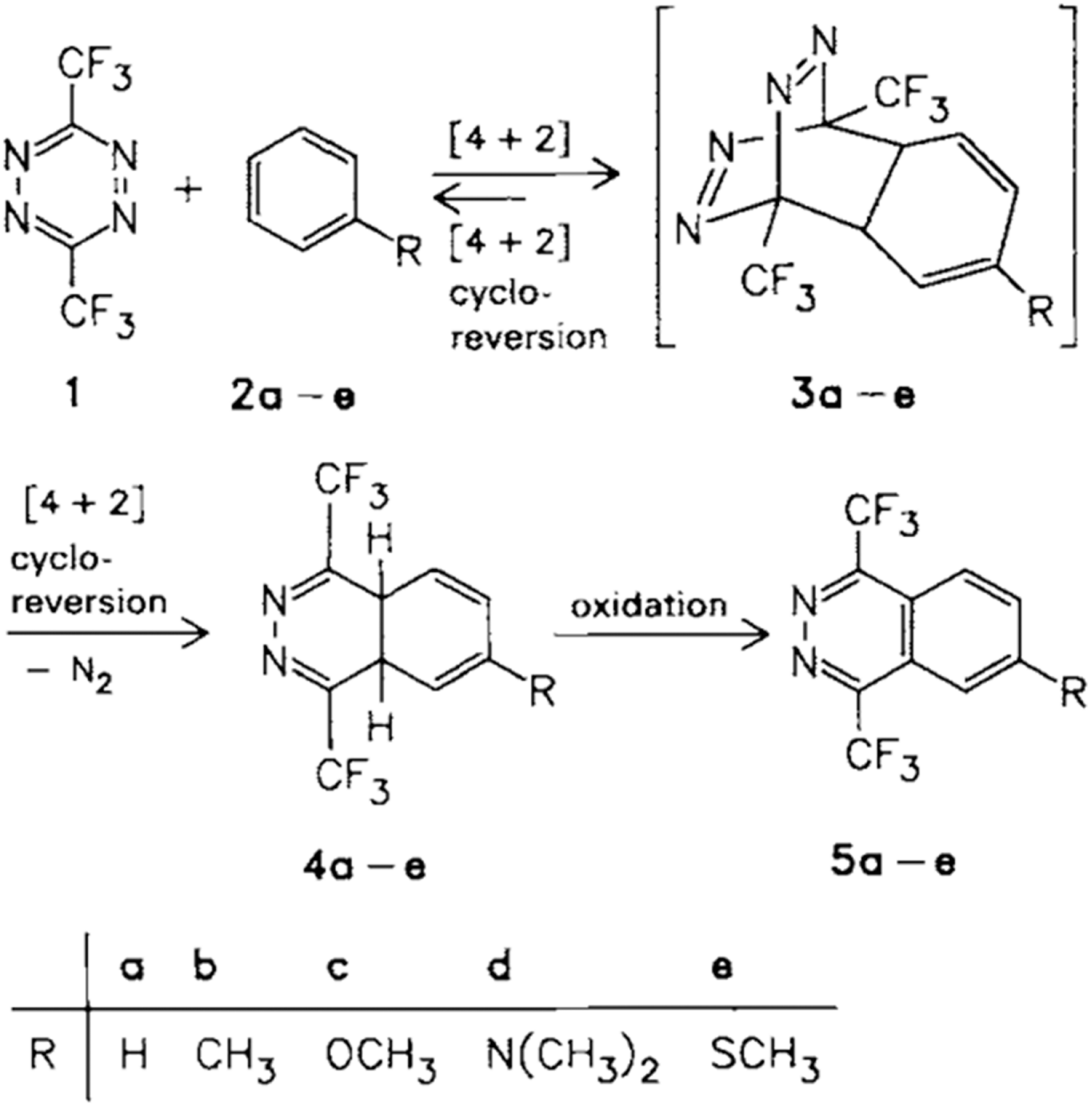
Proposed mechanism of the Diels–Alder reaction between bis­(trifluoromethyl)­tetrazine and different benzene derivatives. With permission reproduced from Seitz et al.^12^ Copyright 1987 by VCH Verlagsgesellschaft mbH, Germany.

The same intrinsic high Diels–Alder reactivity of 1,2,4,5-tetrazines has driven extensive interest in chemistry over the past decades. In a seminal study, Sauer and co-workers demonstrated that these aromatic dienes undergo exceptionally fast inverse-electron-demand Diels–Alder (IEDDA) reactions.^13^ Subsequently, Blackman et al. showed that reactions between tetrazines and strained alkenes such as *trans*-cyclooctene proceed as exceptionally rapid bioorthogonal cycloadditions.^14^ This reaction has since become a cornerstone of bioorthogonal chemistry, finding applications in biomolecular labeling,^15,16^ radiolabeling and pretargeted imaging,^17,18^ as well as in targeted drug-delivery systems.^19^ Although the reaction of tetrazines with benzene derivatives is not suitable for such applications due to the harsh conditions required, it provides an interesting synthetic entry to phthalazine derivatives.

In the work by Seitz and co-workers, benzene and electron-rich benzene derivatives were reacted with the tetrazine in solutions of the respective arene at temperatures of up to 140 °C for 12–24 h, affording yields between 40 and 87% (Table 1). The proposed mechanism is shown in Figure 1 and is consistent with the now established mechanism for Diels–Alder reactions of 1,2,4,5-tetrazines.^13^

**Table 1:** Reaction Conditions and Yields of the Diels–Alder Reactions Reported by Seitz and Co-Workers^12^

compound	original label	dienophile	reaction conditions	yield [%]^a^
**1**	**2a**	benzene	24 h, 140 °C	87
**2**	**2b**	toluene	24 h, 140 °C	40
**3**	**2c**	anisole	12 h, 140 °C	68
**4**	**2e**	thioanisole	24 h, 140 °C	56
**5**	**2d**	*N,N*-dimethylaniline	12 h, 110 °C	57

a Yields are reported with respect to products **5a**–**e**.

However, despite the experimental demonstration of this unusual reactivity, the mechanistic details of the tetrazine–benzene cycloaddition have not yet been investigated in depth. In particular, the origins of benzene’s ability to act as a dienophile under these conditions remain unclear. Here, we present a computational study that elucidates the reaction mechanism and examines the factors governing this exceptional reactivity, including the influence of tetrazine substitution.

## Methodology

DFT calculations were performed using ORCA 6.0.0 (Windows 64-bit)^20,21^ at the DLPNO–CCSD­(T)/cc-pVTZ//M06–2X/def2-TZVP level of theory.^22^ Solvation effects were included using the Conductor-like Polarizable Continuum Model (CPCM). For each reaction, the dienophile was used as a solvent, except in the case of norbornene where benzene was used. For benzene, toluene, and anisole, default solvent models were used. Thioanisole and *N,N*-dimethylaniline were defined using their dielectric constants and refractive indices (values are provided in the Supporting Information Table S1). The RIJCOSX approximation^23^ was used to accelerate calculations. Thermodynamic values were calculated at 298.15, 383.15, and 413.15 K but are discussed at 413.15 K in this manuscript. Frontier molecular orbital energies were extracted from the DLPNO–CCSD­(T)/cc-pVTZ single-point calculations including CPCM solvation described above. Hirshfeld charge analyses were performed at the DLPNO–CCSD­(T)/cc-pVTZ level of theory, using identical solvation models.

Energy Decomposition Analysis (EDA) was performed using the Extended Transition State formalism in ORCA 6.1.0 (Windows 64bit) at the M06–2X/def2-TZVP level of theory in the gas phase.^24^ Δ*E*_XC_^0^ is summed into Δ*E*_Pauli_ as proposed by Ziegler and co-workers.^25^ Orbital overlap values between the dominant interacting frontier orbitals were calculated using Multiwfn 3.7.^26^ The overlaps were obtained from fragment orbital wave functions at the M06–2X/def2-TZVP level of theory.

All calculations were performed on a Lenovo IdeaCentre AIO 3 27ALC6 equipped with an AMD Ryzen 7 7730u CPU and 16 GB of DDR4 RAM @3200 MHz running Windows 11.

Visualization of 3D structures was achieved using the ChemView platform.^27^

## Results and Discussion

We first investigated the energy profile of the reaction between benzene (**1**) and 3,6-bis­(trifluoromethyl)-1,2,4,5-tetrazine (**CF**_**3**_ **Tz**) (Figure 2). The calculated reaction profile is consistent with the mechanism proposed by Seitz and co-workers (Figure 1). The transition state (TS) for the initial cycloaddition has an activation free energy of 42.5 kcal/mol at 140 °C, with a corresponding activation enthalpy of 21.6 kcal/mol. While the free energy barrier appears high, the reaction was experimentally carried out in neat solvent at elevated temperature and pressure in an autoclave, such that entropic penalties are partially mitigated. The computed Δ*G* ^‡^ slightly overestimates the effective experimental barrier but remains compatible with feasible reactivity. This first cycloaddition step constitutes the rate-limiting step of the reaction. The initial Diels–Alder intermediate lies at Δ*G* = 22.2 kcal/mol and in theory enables a reversible retro-Diels–Alder reaction with a barrier of Δ*G* ^‡^ = 20.3 kcal/mol. However, the competing retro-Diels–Alder pathways accompanied by dinitrogen extrusion (**TS2**_**benzene**_ and **TS3**_**benzene**_) are significantly lower in energy (Δ*G* = 33.2 and 31.5 kcal/mol; Δ*G* ^‡^ = 11.0 and 9.3 kcal/mol for **TS2**_**benzene**_ and **TS3**_**benzene**_, respectively), thereby allowing rapid conversion to product **P**_**benzene**_ (Δ*G* = −36.8 kcal/mol). This is in overall agreement with the originally proposed mechanism; however, our calculations indicate direct conversion from the starting materials to the cycloaddition intermediate, rather than a discrete equilibrium between starting material and intermediate.

**Figure 2:**
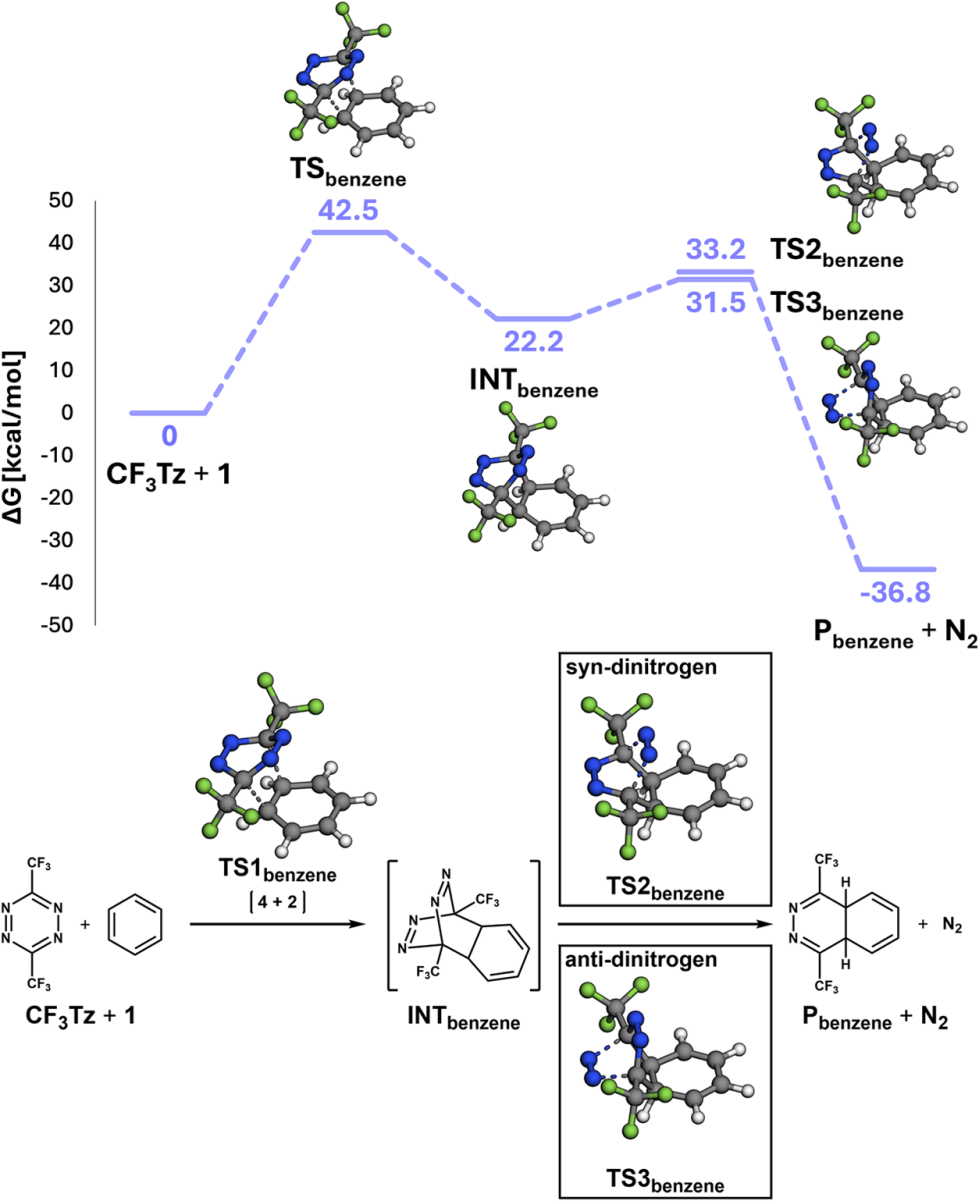
Free energy profile and scheme of the reaction between **1** and **CF**_**3**_ **Tz**. Values are in kcal/mol.

As illustrated in Figure 2, dinitrogen extrusion can proceed via syn and anti transition states, which differ in the relative orientation of the departing N_2_ fragment with respect to the newly formed ring system. A preference for extrusion of anti-dinitrogen (**TS3**_**benzene**_) over syn-dinitrogen (**TS2**_**benzene**_) of approximately 2 kcal/mol is observed. This preference was previously also noted by Houk and co-workers for cyclopropene–tetrazine cycloadditions;^28^ however, they also demonstrated that dynamic effects can invert this preference and accelerate the retro-Diels–Alder reaction.

Next, we investigated the effect of benzene substitution on the rate-limiting IEDDA step. We considered the same arene derivatives examined by Seitz et al.: toluene (**2**), anisole (**3**), thioanisole (**4**), and *N,N*-dimethylaniline (**5**). Calculated activation electronic energies (Δ*E* ^‡^), enthalpies (Δ*H* ^‡^), and free energies (Δ*G* ^‡^) for the reaction with **CF**_**3**_ **Tz** at 140 °C are summarized in Table 2. The computed barriers show a general tendency to decrease with increasing arene electron richness and are in qualitative agreement with the experimentally reported yields, temperatures, and reaction times by Seitz et al., despite the limited quantitative precision of the experimental kinetic data. The overall trend is broadly consistent with frontier molecular orbital (FMO) theory, in which donation raises the arene π-HOMO level, reducing the HOMO–LUMO+1 gap and enhancing orbital interactions with the tetrazine acceptor orbitals. Notably, however, thioanisole does not follow this trend: it exhibits a higher HOMO energy than anisole (−8.15 eV vs −8.54 eV) while also displaying a higher activation barrier (−41.1 kcal/mol vs −39.7 kcal/mol). This discrepancy indicates that FMO considerations alone are insufficient to account for the observed reactivity differences.

**Table 2:** DLPNO–CCSD­(T)//M06-2X Calculated Electronic Energies (Δ*E* ^‡^), Enthalpies (Δ*H* ^‡^), and Free Energies (Δ*G* ^‡^) for the Reaction of **1–5** with **CF**_**3**_ **Tz** at 140 °C in kcal/mol, Together with the Corresponding Arene HOMO Energies (*E*_HOMO_, in eV) Extracted from the DLPNO–CCSD­(T) Single-Point Calculations

compound	dienophile	Δ*E* ^‡^	Δ*H* ^‡^	Δ*G* ^‡^	*E*_HOMO_
**1**	benzene	20.9	21.6	42.5	–9.19
**2**	toluene	19.0	19.7	41.5	–8.84
**3**	anisole	17.2	17.9	39.7	–8.54
**4**	thioanisole	18.5	19.1	41.1	–8.15
**5**	*N,N*-dimethylaniline	10.2	11.1	32.9	–7.65

While most arenes follow the expected reactivity trend, two reactions warrant closer examination. In particular, thioanisole exhibits a higher activation barrier than expected based on its elevated HOMO energy, and *N,N*-dimethylaniline (**5**) displays an unusually high reactivity compared to the rest of the series.

To rationalize these reactivities, we first investigated possible solvation effects. All systems were calculated both in the gas phase and with CPCM solvation corresponding to the experimental conditions in the original work by Seitz et al. Relative reactivity trends remain consistent between the two environments (see Supporting Information Figure S1). Solvation stabilization, defined as the difference in activation free energies between the gas-phase and solvated calculations, is shown in Figure 4. Solvation has only a minor effect on arenes **1**–**4**, lowering the activation barriers by less than 1 kcal/mol. In contrast, *N,N*-dimethylaniline (**5**) exhibits a significantly larger stabilization of 5.3 kcal/mol, clearly distinguishing it from the rest of the series.

To rationalize this behavior, the transition state must be considered. The transition state of **5** with **CF**_**3**_ **Tz** is highly asynchronous (Figure 3). In inverse-electron-demand Diels–Alder reactions, bond formation arises from interaction between the arene HOMO and the tetrazine acceptor orbitals. When this interaction is spatially uneven, bond formation becomes asynchronous. Electron-rich arenes exhibit stronger and more polarized interactions, resulting in greater imbalance between forming bonds and increased charge transfer. For example, Hirshfeld charge analysis (Figure 3) shows increased charge separation for *N,N*-dimethylaniline (0.564 e) compared to benzene (0.256 e). However, the relationship between asynchronicity and solvent stabilization is not linear. For arenes **1**–**4**, solvation lowers the activation free energies only weakly despite differences in transition state asynchronicity. In contrast, *N,N*-dimethylaniline exhibits a pronounced increase in solvation stabilization, suggesting a strongly nonlinear increase in solvent stabilization at high degrees of charge separation. While this rationalizes the exceptional reactivity of *N,N*-dimethylaniline, it does not account for the anomalous behavior of thioanisole, indicating that additional factors beyond simple FMO considerations and polarity-driven solvation contribute to the observed reactivity. Additionally, increased charge separation alone is insufficient to account for the observed solvent effects.

**Figure 3:**
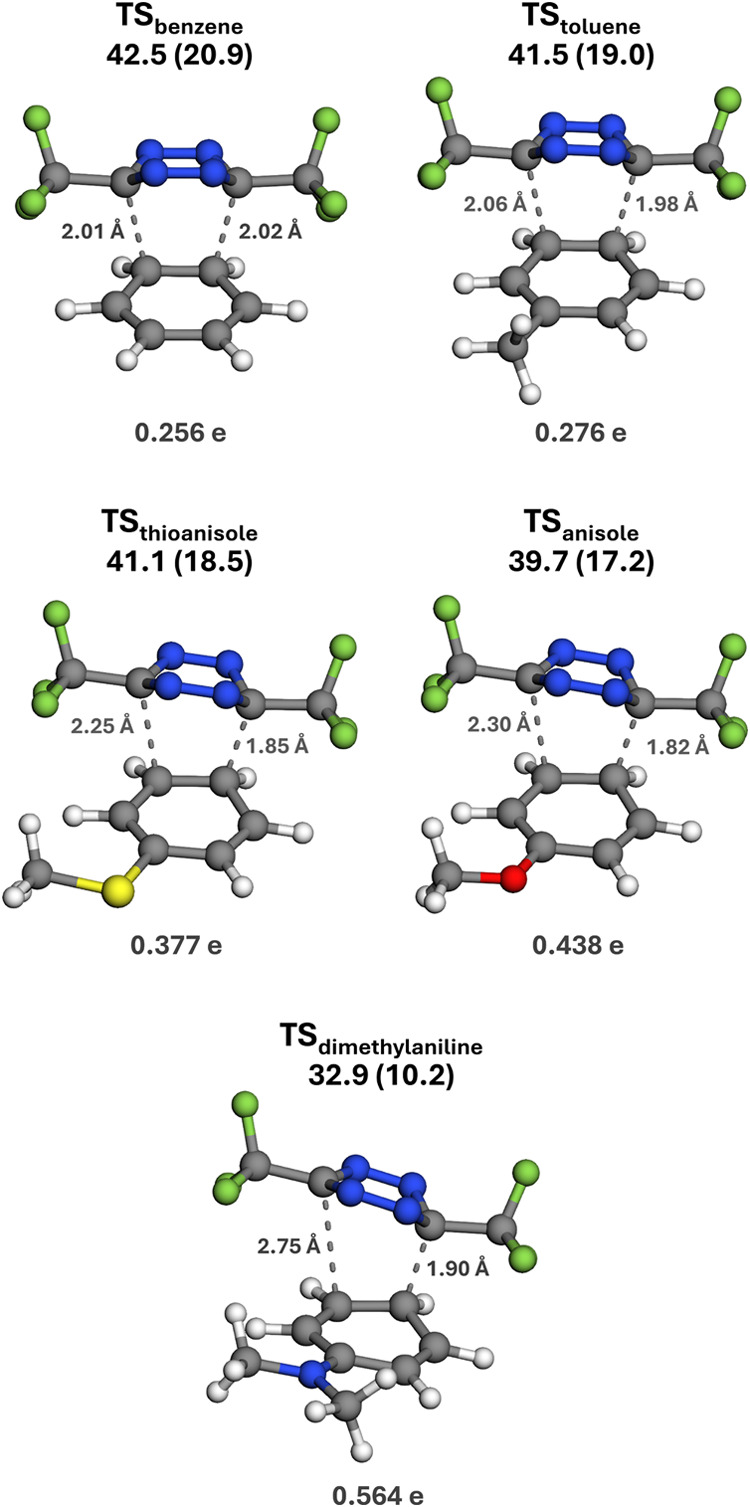
Transition state geometries between arenes **1–5** and **CF**_**3**_ **Tz**. Gibbs free energy (Δ*G* ^‡^) as well as electronic energy (Δ*E* ^‡^) (shown in parentheses) at 140 °C given in kcal/mol. Hirshfeld molecular charge (*Q* ^H^) for each arene at the transition state given in units of e.

**Figure 4:**
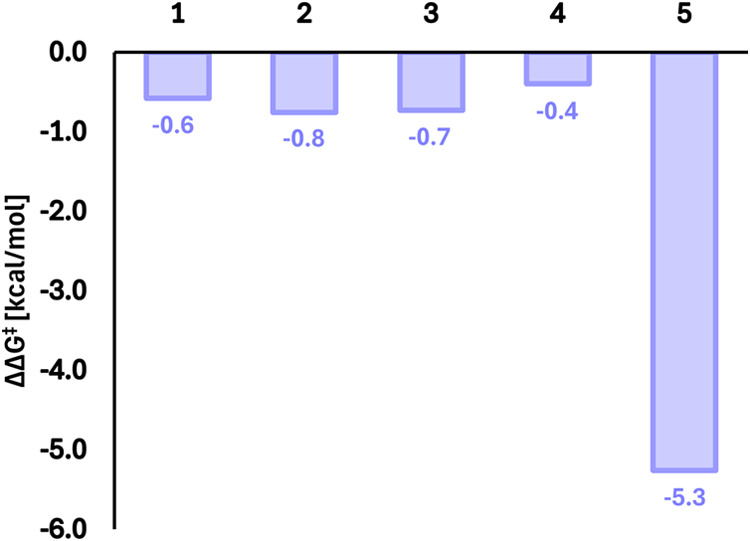
Activation free energies in solution relative to gas phase at 140 °C in kcal/mol.

To further analyze the reactivity of these systems and the origin of the pronounced asynchronicity observed for *N,N*-dimethylaniline, we performed a Distortion/Interaction (DI) analysis in combination with Energy Decomposition Analysis (EDA) for all arene–tetrazine systems. In the DI framework, Δ*E* of an interacting complex, such as a transition state, is partitioned into a distortion term Δ*E*_dist_ and an interaction term Δ*E*_int_. The distortion term reflects the energy required to deform the isolated reactants to their transition state geometries, whereas the interaction term corresponds to the energy gained upon bringing these distorted fragments together.^29^

Δ*E*_int_ can further be decomposed using Energy Decomposition Analysis. Here, we employ the Rauk–Ziegler EDA within the Extended Transition State framework as implemented in ORCA 6.1.1.^24^ In this scheme, Δ*E*_int_ is partitioned into three physically meaningful contributions: Δ*V*_elec_, which accounts for the classical electrostatic interaction between the unperturbed fragment charge distributions; Δ*E*_Pauli_, which represents the destabilizing Pauli repulsion arising from occupied–occupied orbital overlap; and Δ*E*_OI_, which describes the stabilizing orbital interaction associated with charge transfer and polarization between the fragments. These analyses were performed in the gas phase to isolate intrinsic electronic effects, as solvation effects were evaluated separately.

The transition states exhibit varying degrees of asynchronicity (Figure 3) and differ in their position along the reaction coordinate. Because DI/EDA results depend strongly on the chosen geometry, particularly on the distance between the reactants (i.e., forming bond distances), direct comparison of transition states located at different positions along the reaction coordinate can limit interpretability.^29^ To address this issue, DI/EDA is commonly performed along a reaction coordinate,^30–34^ allowing comparison at equivalent forming bond distances and enabling a consistent interpretation of the factors contributing to the activation barrier. However, this approach requires the definition of a suitable reaction coordinate. In cycloadditions with varying degrees of asynchronicity, the choice of coordinate is less straightforward and may influence the resulting interpretation.

An alternative and often used strategy employs a “consistent geometry” approach, which can be viewed as a simplified form of reaction-coordinate analysis.^35^ In this method, representative bond distances (e.g., average forming bond lengths at the transition states) are imposed for all systems. Although this procedure generates slightly artificial transition state geometries, it enables direct comparison of electronic and steric effects across the series. Because this approach removes effects arising from differing degrees of asynchronicity, it is well suited for systems such as those investigated in this work. This approach enables a controlled comparison of interaction terms across different systems.

However, a single analysis at a fixed geometry does not explain how asynchronicity arises. In principle, this could be addressed by analyzing the full range of forming bond distances and degrees of asynchronicity through construction of a two-dimensional energy surface. Although such an approach has recently been introduced,^36,37^ it has not yet been extended to enable detailed DI/EDA analysis of asynchronicity. Therefore, the “consistent geometry” approach remains commonly employed, but the analysis is performed at multiple representative geometries corresponding to different degrees of asynchronicity. This allows trends to be identified and provides insight into the origin of the observed behavior.^38,39^

In this work, two defined ”consistent geometries” were used. First, synchronous reference transition state geometries (**synch-TS**) were constructed by constraining both forming bond lengths to the same value (2.00 Å). Second, asynchronous constrained TS geometries (**asynch-TS**) were generated in which both forming bond lengths were fixed to predefined but unequal values of 2.75 Å and 1.90 Å, thereby imposing an identical degree of asynchronicity across all systems. The trends observed are consistent across both imposed geometries and agree with those obtained from the optimized transition states, indicating that the conclusions are not an artifact of the applied constraints but reflect intrinsic electronic factors contributing to the observed reactivity. For reference, EDA calculations on the unconstrained TS geometries are provided in the Supporting Information (Table S11).

In addition to the systems studied by Seitz and co-workers, we analyzed the reaction between norbornene (**Nor**) and 3,6-dimethyl-1,2,4,5-tetrazine (**DMT**) as an experimentally established click reaction.^40^ This reaction is well documented in the literature and proceeds under mild conditions (ambient temperature in dichloromethane), albeit at a slower rate than more activated strained alkene-tetrazine systems reported in click-chemistry. It provides a useful reference for the absolute magnitude of the energy components obtained from our EDA, serving as a benchmark against an experimentally validated system rather than for direct comparison of reaction rates.

Table 3 summarizes the DI/EDA results for the synchronous TS case. Comparison of **synch-TS**_**benzene**_ (2.00 Å) and **synch-TS**_**Nor/DMT**_, both of which naturally adopt synchronous transition states, shows that the higher reactivity of the latter arises from a significantly more favorable interaction energy. This difference is driven by a more stabilizing Δ*E*_OI_ (−116 vs −101 kcal/mol) and Δ*V*_elstat_ (−204 vs −189 kcal/mol), while Δ*E*_Pauli_ disfavor the **Nor/DMT** reaction. The HOMO energies of norbornene and benzene are very similar (−9.16 eV vs −9.19 eV, respectively), whereas the LUMO+1 of **CF**_**3**_ **Tz** (−12.26 eV) is significantly stabilized relative to that of **DMT** (−10.67 eV). As a result, the HOMO–LUMO+ 1 gap is smaller for the **Nor/DMT** system, in line with the more stabilizing orbital interaction Δ*E*_OI_ observed. However, Δ*E*_OI_ is not only influenced by orbital gaps Δε, but also overlap S (eq 1).

1 $$\Delta E_{\mathrm{OI}}\propto -\frac{S^{2}}{\Delta \varepsilon }$$

**Table 3:** Energy Decomposition Analysis of **synch-TS** (2.0 Å) for the Reactions of **1–5** with **CF**_**3**_ **Tz** and **Nor** with **DMT** ^a^

	benzene	toluene	anisole	thioanisole	*N,N*-dimethylaniline	Nor/DMT
Δ*E* ^‡^	**23.6**	**21.7**	**20.1**	**21.0**	**19.2**	**6.0**
Δ*E*_dist_	**51.0**	**47.5**	**56.4**	**56.4**	**56.1**	**55.3**
Δ*E*_int_	–**27. 4**	–**25. 7**	–**36. 4**	–**35. 4**	–**36. 9**	–**49. 3**
Δ*E*_OI_	–101.1	–95.1	–111.6	–111.1	–111.8	–115.7
Δ*V*_elstat_	–189.1	–183.8	–200.6	–197.6	–205.9	–203.7
Δ*E*_Pauli_	262.4	252.8	275.5	273.0	280.5	270.2

a All values are shown in kcal/mol.

To quantify this effect, we computed the overlap between the dominant interacting frontier orbitals for representative systems. The **1**/**CF**_**3**_ **Tz** pair exhibits a small overlap (*S*_1/CF_3_Tz_ = 0.07), whereas the **Nor/DMT** system shows a substantially larger overlap (*S*_Nor/DMT_ = 0.27). This increase in overlap further enhances the stabilizing Δ*E*_OI_ in the norbornene system. This difference arises from the fundamentally different nature of the underlying π-systems. The π-HOMO of benzene is delocalized over six carbon atoms, whereas the π orbital of norbornene is localized at the reacting double bond, leading to larger coefficients at the reactive centers. Consequently, both a smaller orbital energy gap and increased overlap contribute to the stronger Δ*E*_OI_ and higher reactivity of norbornene. On this basis, benzene and its derivatives can be regarded as intrinsically less reactive dienophiles. While this analysis highlights the importance of orbital overlap for the comparison between benzene and norbornene, differences within the substituted benzene series arise from substituent-induced changes in electronic structure, which affect both the HOMO energies and the balance of interaction terms revealed by DI/EDA.

Comparing different benzene derivatives, aryl reactivity in both the synchronous and asynchronous artificial transition states follows the same trend as in the fully optimized unconstrained transition state. Interestingly, in the synchronous transition state, orbital interactions alone do not account for the observed reactivity differences; instead, electrostatic interactions contribute significantly. In Klopman’s terminology, this corresponds to a charge-controlled reaction.^41^ Such a “polar mechanism” has previously been identified for Diels–Alder reactions,^42^ and electrostatics has been shown to govern reactivity differences in several related cases.^43–45^

However, only benzene and toluene exhibit an (almost) synchronous transition state. Comparing the EDA values for these two reactants shows that the higher reactivity of toluene arises from increased orbital and electrostatic interactions in agreement with frontier molecular orbital theory and the charge-controlled reactivity concepts. Thioanisole and anisole both adopt asynchronous transition states with similar degrees of asynchronicity (see Figure 3). Therefore, performing EDA at a common degree of asynchronicity (Table 4) allows a meaningful comparison of their reactivity. Thioanisole exhibits a higher barrier compared to anisole due to a weaker interaction energy, primarily resulting from reduced electrostatic interactions. This is in agreement with the reduced charge transfer (Figure 3) and explains the non-FMO reactivity trend.

**Table 4:** Energy Decomposition Analysis of **asynch-TS** (2.75 Å and 1.90 Å) for the Reactions of **1–5** with **CF**_**3**_ **Tz** and **Nor** with **DMT** ^a^

	benzene	toluene	anisole	thioanisole	*N,N*-dimethylaniline	Nor/DMT
Δ*E* ^‡^	**25.2**	**22.7**	**19.6**	**20.7**	**14.5**	**20.6**
Δ*E*_dist_	**28.7**	**29.8**	**33.9**	**33.2**	**40.0**	**29.6**
Δ*E*_int_	–**3.5**	–**7.2**	–**14.4**	–**12.5**	–**25.6**	–**9.0**
Δ*E*_OI_	–67.3	–69.9	–75.7	–74.3	–83.9	–66.2
Δ*V*_elstat_	–149.9	–152.3	–155.6	–152.0	–160.8	–153.9
Δ*E*_Pauli_	213.3	214.8	216.8	213.6	219.1	211.0

a All values are shown in kcal/mol.

*N,N*-Dimethylaniline, the most reactive arene studied, derives its high reactivity from stronger interaction and electrostatic contributions in the asynchronous transition state. These contributions significantly lower the activation barrier. While it is also the most reactive substrate in the synchronous transition state geometry (Table 3), the difference is less pronounced. Transitioning to an asynchronous transition state lowers the barrier primarily through a reduction in distortion energy, as previously observed for related tetrazine Diels–Alder reactions.^39^

Adopting an asynchronous geometry also weakens the total interaction energy by reducing both stabilizing (Δ*E*_OI_, Δ*V*_elstat_) and destabilizing (Δ*E*_Pauli_) contributions across the studied reactions. However, in the case of *N,N*-dimethylaniline, the decrease in orbital interaction and electrostatic contributions is substantially smaller than for compounds **1–4**. For those substrates, the loss in interaction energy offsets the gain from reduced distortion. The preferred degree of synchronicity therefore emerges from the balance between loss of orbital interaction and gain in reduced distortion and electrostatic stabilization. For more electron-donating substituents, the latter contributions become increasingly favorable due to stronger polarization and charge-transfer character. For *N,N*-dimethylaniline Δ*E* is lowered considerably because the orbital interaction and electrostatic stabilization remain comparatively strong even at a high degree of asynchronicity, consistent with its higher HOMO energy and enhanced charge-transfer character discussed above. This balance explains the pronounced preference for a highly asynchronous transition state and the overall high reactivity of *N,N*-dimethylaniline.

As this reaction provides an interesting synthetic route to phthalazines, we also investigated whether other tetrazines could participate in this transformation. The **Nor/DMT** results discussed above suggest that only highly reactive tetrazines are capable of undergoing this reaction. Accordingly, calculations were performed using dimethyl 1,2,4,5-tetrazine-3,6-dicarboxylate (**COOCH**_**3**_ **Tz**)^46^ as an alternative electron-deficient tetrazine, and 3,6-diphenyl-1,2,4,5-tetrazine (**PhTz**) as a representative tetrazine commonly employed in bioorthogonal click chemistry. Both were examined in reactions with benzene and *N,N*-dimethylaniline, the corresponding transition state geometries and the activation energies are shown in Figure 5.

**Figure 5:**
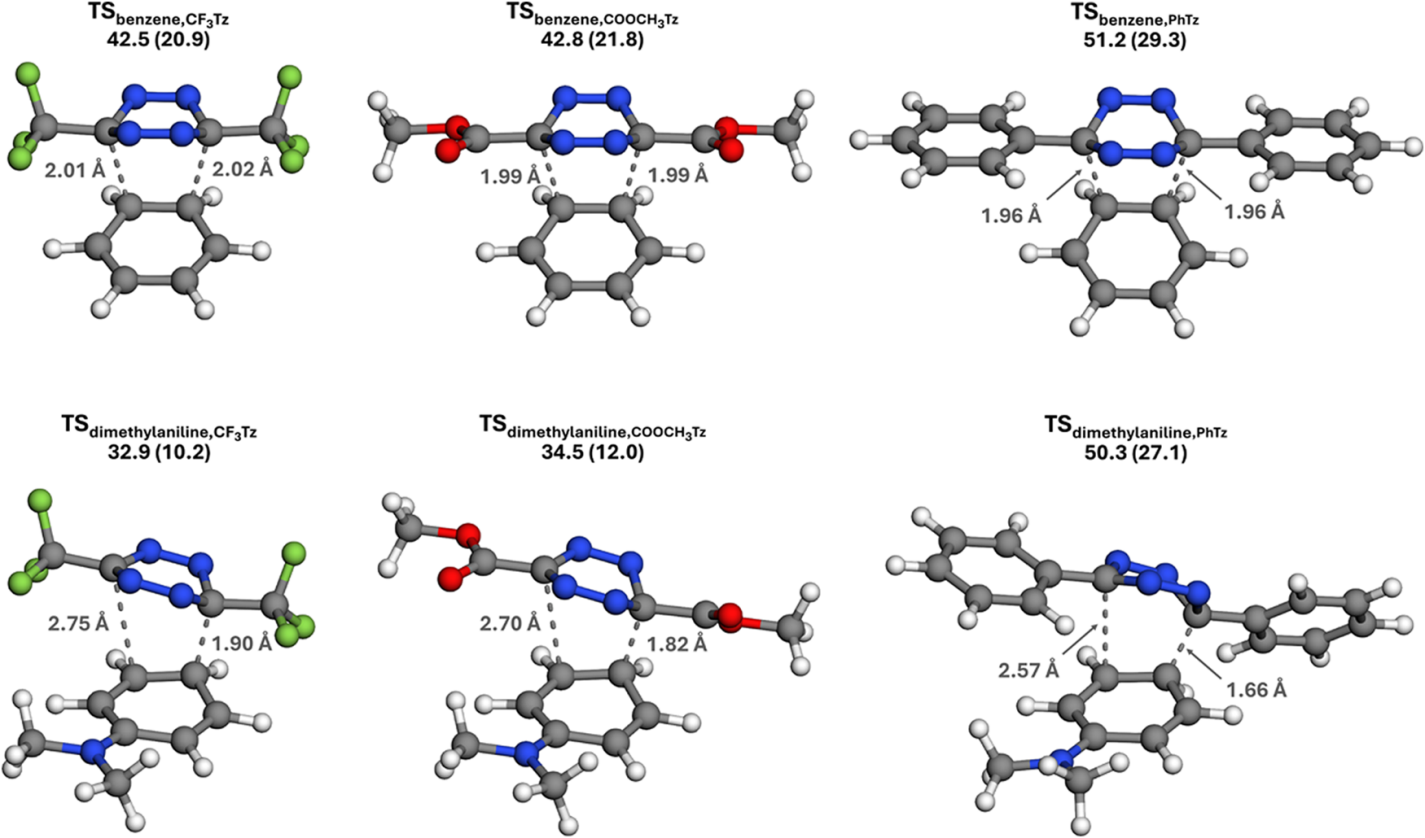
Transition state geometries of benzene as well as *N,N*-dimethylaniline with three different tetrazines: bis­(trifluoromethyl)­tetrazine (**CF**_**3**_ **Tz**), dimethyl 1,2,4,5-tetrazine-3,6-dicarboxylate (**COOCH**_**3**_ **Tz**), and 3,6-diphenyl-1,2,4,5-tetrazine (**PhTz**). Gibbs free energy of activation (Δ*G* ^‡^) as well as electronic energy of activation (Δ*E* ^‡^) (in parentheses) at 140 °C given in kcal/mol.

As summarized in Table 5, **COOCH**_**3**_ **Tz** is slightly less reactive than **CF**_**3**_ **Tz**, but still exhibits barriers consistent with viable reactivity. In contrast, **PhTz** displays substantially higher activation barriers, rendering the reaction inaccessible under comparable conditions for the reaction with both benzene and *N,N*-dimethylaniline.

**Table 5:** DLPNO–CCSD­(T)//M06-2X Calculated Activation Energies at 140 °C in kcal/mol

		Δ*E* ^‡^	Δ*H* ^‡^	Δ*G* ^‡^
	CF_3_Tz	20.9	21.6	42.5
benzene	COOCH_3_Tz	21.8	22.4	42.8
	PhTz	29.3	29.8	51.2
	CF_3_Tz	10.2	11.1	32.9
*N,N*-dimethylaniline	COOCH_3_Tz	12.0	12.9	34.5
	PhTz	27.1	26.2	50.3

These results demonstrate that only strongly electron-deficient tetrazines are capable of undergoing this reaction, as sufficiently stabilizing interaction energies are required to compensate for the intrinsically poor orbital overlap of the arene dienophile. Accordingly, only the most electron-deficient systems, such as 3,6-bis­(trifluoromethyl)-1,2,4,5-tetrazine and dimethyl 1,2,4,5-tetrazine-3,6-dicarboxylate, exhibit viable reactivity.

## Conclusion

We computationally investigated one of the rare examples of benzene acting as a dienophile in arene–tetrazine cycloadditions. Our results show that only highly activated tetrazines are capable of undergoing this reaction, which is intrinsically disfavored by the compact and poorly overlapping π-orbitals of aromatic systems.

Reactivity of different arenes is governed by a combination of frontier molecular orbital effects and charge-control contributions. Substituent effects influence the activation barrier, in part by enabling shifts toward more asynchronous and polar transition states. In these cases, reduced distortion energy contributes to lowering the barrier, while the accompanying changes in orbital and electrostatic interactions further modulate the overall reactivity. Asynchronicity therefore does not act as an independent driving force, but rather alters the balance between these contributions along the reaction pathway.

Overall, this study provides mechanistic insight into a rare class of cycloadditions and clarifies the electronic factors that enable arene participation in tetrazine Diels–Alder reactions.

## Supplementary Material



## Data Availability

Output files are archived at 10.5281/zenodo.18760736.

## Notes

Additional computational details, computational data (PDF)
