# Supplementary material for: Insights into Tetrazine–Benzene Cycloadditions
Source: J Phys Chem A. 2026 Jun 3;130(24):4545–52. doi: 10.1021/acs.jpca.6c01346 (PMC13288704; doi:10.1021/acs.jpca.6c01346)
Supplement: Supplementary file 1 [file jp6c01346_si_001.pdf]

Supporting Information

Insights into Tetrazine–Benzene Cycloadditions

Tori Demuth[1] and Dennis Svatunek*[1]

[1]Institute of Applied Synthetic Chemistry, TU Wien, Getreidemarkt 9, 1060 Vienna, Austria

*Email: dennis.svatunek@tuwien.ac.at

Contents

Methods **S2**

Data **S3**

Geometry Optimization (M06-2X/def2-TZVP) S3

Single-Point Correction (DLPNO-CCSD(T)/cc-pVTZ//M06-2X/def2-TZVP) S7

Energy Decomposition Analysis S9

Comparison of Different Tetrazines S10

Methods

For non-predefined solvents, CPCM parameters were specified manually in ORCA using experimental values for the dielectric constant (ε) and refractive index (n).

The manually specified solvent parameters are summarized in Table S1.

Table S1: Manually specified CPCM parameters for non-predefined solvents.

Solvent	Refractive Index (n)	Dielectric Constant (ε)
thioanisole	1.58	4.76
N,N-dimethylaniline	1.55	4.40

Data

Geometry Optimization (M06-2X/def2-TZVP)

Table S2: M06-2X/def2-TZVP energies used for the Diels–Alder reaction between **benzene** (**1**) and **CF_3Tz**, given in Eh.

		solvent (benzene)			no solvent		
		298.15 K	383.15 K	413.15 K	298.15 K	383.15 K	413.15 K
	ΔE		−232.2277308			−232.2252052	
**$SM_{benzene}$**	ΔH	−232.1181578	−232.1169129	−232.1169129	−232.1155968	−232.1143513	−232.1143513
	ΔG	−232.1532352	−232.1627549	−232.1662934	−232.1506801	−232.1602012	−232.1637403
	ΔE		−970.4839469			−970.4787101	
**SM_{CF_3Tz}**	ΔH	−970.4040509	−970.4016983	−970.4016983	−970.3986865	−970.3963365	−970.3963365
	ΔG	−970.4598599	−970.4743144	−970.4797420	−970.4553326	−970.4700189	−970.4755278
	ΔE		−1202.673636			−1202.666377	
**$TS_{benzene}$**	ΔH	−1202.483157	−1202.479483	−1202.479483	−1202.475641	−1202.471974	−1202.471974
	ΔG	−1202.550056	−1202.567043	−1202.573548	−1202.542346	−1202.559299	−1202.565792
	ΔE		−1202.702978			−1202.696337	
**$INT_{benzene}$**	ΔH	−1202.510225	−1202.506518	−1202.506518	−1202.503352	−1202.499651	−1202.499651
	ΔG	−1202.576459	−1202.593282	−1202.599735	−1202.569529	−1202.586342	−1202.592790
	ΔE		−1202.683218				
**$TS2_{benzene}$**	ΔH	−1202.492828	−1202.489135	−1202.489135			
	ΔG	−1202.559546	−1202.576501	−1202.583000			
	ΔE		−1202.684673				
**$TS3_{benzene}$**	ΔH	−1202.494132	−1202.490439	−1202.490439			
	ΔG	−1202.561105	−1202.578119	−1202.584638			
	ΔE		−1093.228387				
**$P_{benzene}$**	ΔH	−1093.048255	−1093.044859	−1093.044859			
	ΔG	−1093.112722	−1093.129123	−1093.135380			
	ΔE		−109.5362623				
**N_2**	ΔH	−109.5262424	−109.5259090	−109.5259090			
	ΔG	−109.5495607	−109.5560629	−109.5584105			

Table S3: M06-2X/def2-TZVP energies used for the Diels–Alder reaction between **toluene** (**2**) and **CF_3Tz**, given in Eh.

		solvent (toluene)			no solvent		
		298.15 K	383.15 K	413.15 K	298.15 K	383.15 K	413.15 K
**$SM_{toluene}$**	ΔE		−271.5384187			−271.5357108	
	ΔH	−271.3986849	−271.3970932	−271.3970932	−271.3958696	−271.3942784	−271.3942784
	ΔG	−271.4391801	−271.4500370	−271.4540955	−271.4363545	−271.4472126	−271.4512717
**SM_{CF_3Tz}**	ΔE		−970.4841435			−970.4787101	
	ΔH	−970.4042573	−970.4019044	−970.4019044	−970.3986865	−970.3963365	−970.3963365
	ΔG	−970.4598542	−970.4742472	−970.4796532	−970.4553326	−970.4700189	−970.4755278
**$TS_{toluene}$**	ΔE		−1241.987437			−1241.979731	
	ΔH	−1241.766800	−1241.762781	−1241.762781	−1241.758756	−1241.754744	−1241.754744
	ΔG	−1241.837854	−1241.855795	−1241.862683	−1241.829598	−1241.847505	−1241.854381
**$INT_{toluene}$**	ΔE		−1242.016557			−1242.009632	
	ΔH	−1241.793627	−1241.789576	−1241.789576	−1241.786401	−1241.782357	−1241.782357
	ΔG	−1241.864071	−1241.881871	−1241.888716	−1241.856805	−1241.874601	−1241.881443

Table S4: M06-2X/def2-TZVP energies used for the Diels–Alder reaction between **anisole** (**3**) and **CF_3Tz**, given in Eh.

		solvent (anisole)			no solvent		
		298.15 K	383.15 K	413.15 K	298.15 K	383.15 K	413.15 K
**$SM_{anisole}$**	ΔE		−346.7568229			−346.7513979	
	ΔH	−346.6105236	−346.6087644	−346.6087644	−346.6049538	−346.6031969	−346.6031969
	ΔG	−346.6532114	−346.6646161	−346.6688924	−346.6476295	−346.6590306	−346.6633054
**SM_{CF_3Tz}**	ΔE		−970.4859799			−970.4787101	
	ΔH	−970.4061603	−970.4038059	−970.4038059	−970.3986865	−970.3963365	−970.3963365
	ΔG	−970.4617468	−970.4761385	−970.4815443	−970.4553326	−970.4700189	−970.4755278
**$TS_{anisole}$**	ΔE		−1317.211722			−1317.198894	
	ΔH	−1316.984563	−1316.980371	−1316.980371	−1316.971201	−1316.967024	−1316.967024
	ΔG	−1317.057965	−1317.076410	−1317.083496	−1317.044279	−1317.062679	−1317.069750
**$INT_{anisole}$**	ΔE		−1317.230510			−1317.218876	
	ΔH	−1317.000838	−1316.996621	−1316.996621	−1316.988891	−1316.984682	−1316.984682
	ΔG	−1317.073461	−1317.091707	−1317.098728	−1317.061573	−1317.079835	−1317.086860

Table S5: M06-2X/def2-TZVP values for the Diels–Alder reaction between **thioanisole** (**4**) and **CF_3Tz**, given in Eh.

		solvent (thioanisole)			no solvent		
		298.15 K	383.15 K	413.15 K	298.15 K	383.15 K	413.15 K
**$SM_{thioanisole}$**	ΔE		−669.7274209			−669.7215427	
	ΔH	−669.5840332	−669.5821761	−669.5821761	−669.5780844	−669.5762273	−669.5762273
	ΔG	−669.6287623	−669.6406323	−669.6450848	−669.6228431	−669.6347163	−669.6391698
**SM_{CF_3Tz}**	ΔE		−970.4862147			−970.4787101	
	ΔH	−970.4064029	−970.4040484	−970.4040484	−970.3986865	−970.3963365	−970.3963365
	ΔG	−970.4619833	−970.476374	−970.4817795	−970.4553326	−970.4700189	−970.4755278
**$TS_{thioanisole}$**	ΔE		−1640.180319			−1640.167311	
	ΔH	−1639.956287	−1639.951995	−1639.951995	−1639.942777	−1639.938497	−1639.938497
	ΔG	−1640.031490	−1640.050324	−1640.057560	−1640.017760	−1640.036572	−1640.043800
**$INT_{thioanisole}$**	ΔE		−1640.203346			−1640.191544	
	ΔH	−1639.976759	−1639.972437	−1639.972437	−1639.964779	−1639.960461	−1639.960461
	ΔG	−1640.051824	−1640.070624	−1640.077853	−1640.040303	−1640.059205	−1640.066467

Table S6: M06-2X/def2-TZVP energies used for the Diels–Alder reaction between ***N,N*-dimethylaniline** (**5**) and **CF_3Tz**, given in Eh.

		solvent (*N,N*-dimethylaniline)			no solvent		
		298.15 K	383.15 K	413.15 K	298.15 K	383.15 K	413.15 K
**$SM_{dimethylaniline}$**	ΔE		−366.1872482			−366.1815434	
	ΔH	−365.9977462	−365.9955953	−365.9955953	−365.9918325	−365.9896846	−365.9896846
	ΔG	−366.0451639	−366.0576498	−366.0623590	−366.0392140	−366.0516882	−366.0563928
**SM_{CF_3Tz}**	ΔE		−970.4860409			−970.4787101	
	ΔH	−970.4062240	−970.4038695	−970.4038695	−970.3986865	−970.3963365	−970.3963365
	ΔG	−970.4617933	−970.4761798	−970.4815838	−970.4553326	−970.4700189	−970.4755278
**$TS_{dimethylaniline}$**	ΔE		−1336.656183			−1336.634706	
	ΔH	−1336.385463	−1336.380874	−1336.380874	−1336.363382	−1336.358824	−1336.358824
	ΔG	−1336.463590	−1336.483086	−1336.490595	−1336.440270	−1336.459510	−1336.466927
**$INT_{dimethylaniline}$**	ΔE		−1336.658700			−1336.656930	
	ΔH	−1336.386492	−1336.381885	−1336.381885	−1336.383615	−1336.379028	−1336.379028
	ΔG	−1336.463346	−1336.482577	−1336.489998	−1336.459909	−1336.479019	−1336.486396

Table S7: M06-2X/def2-TZVP energies used for the Diels–Alder reaction between norbornene (**Nor**) and 3,6-dimethyl-1,2,4,5-tetrazine **DMT**, given in Eh.

		solvent (benzene)		
		298.15 K	383.15 K	413.15 K
**SM_{Nor}**	ΔE		−272.69962733	
	ΔH	−272.53961226	−272.53574201	−272.53409096
	ΔG	−272.57424821	−272.58459616	−272.58848221
**SM_{DMT}**	ΔE		−374.95811795	
	ΔH	−374.84239958	−374.83817395	−374.83648823
	ΔG	−374.88271566	−374.89442587	−374.89879084
**$TS_{Nor/DMT}$**	ΔE		−647.64131844	
	ΔH	−647.36386953	−647.35569148	−647.35231042
	ΔG	−647.41414971	−647.42926852	−647.43507983

Table S8: M06-2X/def2-TZVP activation energies for the Diels–Alder reactions between arenes **1-5** and **CF_3Tz** as well as between norbornene (**Nor**) and 3,6-dimethyl-1,2,4,5-tetrazine (**DMT**), given in kcal/mol.

		solvent			no solvent		
		298.15 K	383.15 K	413.15 K	298.15 K	383.15 K	413.15 K
**benzene**	$\Delta E^{\ddagger}$		23.9			23.6	
	$\Delta H^{\ddagger}$	28.3	26.0	24.6	24.2	24.3	24.3
	$\Delta G^{\ddagger}$	39.6	43.9	45.5	40.0	44.5	46.1
**toluene**	$\Delta E^{\ddagger}$		22.0			21.8	
	$\Delta H^{\ddagger}$	22.7	22.7	22.7	22.5	22.5	22.5
	$\Delta G^{\ddagger}$	38.4	43.0	44.6	39.0	43.8	45.4
**anisole**	$\Delta E^{\ddagger}$		19.5			19.6	
	$\Delta H^{\ddagger}$	20.2	20.2	20.2	20.4	20.4	20.4
	$\Delta G^{\ddagger}$	35.8	40.4	42.0	36.8	41.6	43.4
**thioanisole**	$\Delta E^{\ddagger}$		20.9			20.7	
	$\Delta H^{\ddagger}$	21.4	21.5	21.5	21.3	21.4	21.4
	$\Delta G^{\ddagger}$	37.2	41.8	43.5	37.9	42.8	44.5
***N,N*-dimethylaniline**	$\Delta E^{\ddagger}$		10.7			16.0	
	$\Delta H^{\ddagger}$	11.6	11.7	11.7	17.0	17.1	17.1
	$\Delta G^{\ddagger}$	27.2	31.8	33.5	34.1	39.0	40.8
**Nor/DMT**	$\Delta E^{\ddagger}$		10.3				
	$\Delta H^{\ddagger}$	11.4	11.4	11.5			
	$\Delta G^{\ddagger}$	26.9	31.2	32.8			

Single-Point Correction (DLPNO-CCSD(T)/cc-pVTZ//M06-2X/def2-TZVP)

Table S9: DLPNO-CCSD(T)/cc-pVTZ electronic energies, given in Eh.

	solvent	no solvent
**$SM_{benzene}$**	−231.8052468	−231.8029644
**SM_{CF_3Tz}**	−969.1457066	−969.1406038
**$TS_{benzene}$**	−1200.917652	−1200.910836
**$INT_{benzene}$**	−1200.953252	−1200.946997
**$TS2_{benzene}$**	−1200.932581	
**$TS3_{benzene}$**	−1200.935210	
**$P_{benzene}$**	−1091.642073	
**N_2**	−109.3727027	
**$SM_{toluene}$**	−271.0481732	−271.0457448
**SM_{CF_3Tz}**	−969.1459049	−969.1406038
**$TS_{toluene}$**	−1240.163836	−1240.156693
**$INT_{toluene}$**	−1240.199146	−1240.192658
**$SM_{anisole}$**	−346.1612951	−346.1562473
**SM_{CF_3Tz}**	−969.1477243	−969.1406038
**$TS_{anisole}$**	−1315.281534	−1315.270212
**$INT_{anisole}$**	−1315.307735	−1315.296753
**$SM_{thioanisole}$**	−668.7837061	−668.7782848
**SM_{CF_3Tz}**	−969.1479595	−969.1406038
**$TS_{thioanisole}$**	−1637.902181	−1637.890740
**$INT_{thioanisole}$**	−1637.933341	−1637.922465
**$SM_{dimethylaniline}$**	−365.5410613	−365.5358119
**SM_{CF_3Tz}**	−969.1477967	−969.1406038
**$TS_{dimethylaniline}$**	−1334.672607	−1334.655044
**$INT_{dimethylaniline}$**	−1334.688191	−1334.685717
**SM_{Nor}**	−272.2209214	
**SM_{DMT}**	−374.3421530	
**$TS_{Nor/DMT}$**	−646.5495595	

Table S10: DLPNO-CCSD(T)/cc-pVTZ corrected activation energies for the Diels–Alder reactions between arenes **1-5** and $\mathbf{CF_3Tz}$ as well as between norbornene (**Nor**) and 3,6-dimethyl-1,2,4,5-tetrazine (**DMT**), given in kcal/mol.

		solvent			no solvent		
		298.15 K	383.15 K	413.15 K	298.15 K	383.15 K	413.15 K
**benzene**	$\Delta E^‡$		20.9			20.5	
	$\Delta H^‡$	25.3	23.1	21.6	21.2	21.3	21.3
	$\Delta G^‡$	36.6	41.0	42.5	36.9	41.5	43.1
**toluene**	$\Delta E^‡$		19.0			18.6	
	$\Delta H^‡$	19.6	19.7	19.7	19.3	19.3	19.3
	$\Delta G^‡$	35.3	39.9	41.5	35.8	40.6	42.3
**anisole**	$\Delta E^‡$		17.2			16.7	
	$\Delta H^‡$	17.9	17.9	17.9	17.5	17.5	17.5
	$\Delta G^‡$	33.5	38.1	39.7	34.0	38.8	40.5
**thioanisole**	$\Delta E^‡$		18.5			17.7	
	$\Delta H^‡$	19.0	19.1	19.1	18.3	18.4	18.4
	$\Delta G^‡$	34.8	39.4	41.1	34.9	39.8	41.5
**N,N-dimethylaniline**	$\Delta E^‡$		10.2			13.4	
	$\Delta H^‡$	11.1	11.1	11.1	14.4	14.4	14.4
	$\Delta G^‡$	26.7	31.3	32.9	31.4	36.4	38.2
**Nor/DMT**	$\Delta E^‡$		8.5				
	$\Delta H^‡$	9.6	9.6	9.6			
	$\Delta G^‡$	25.0	29.4	30.9			

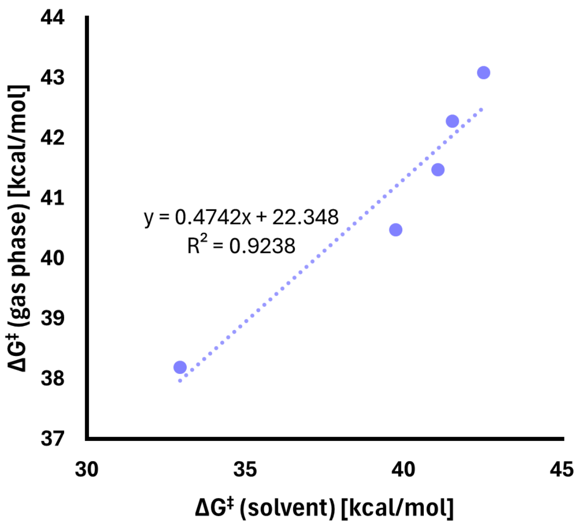

Figure S1: Correlation between $\Delta G^‡$ values in the gas phase and in solution for reactions of $\mathbf{CF_3Tz}$ with compounds **1–5**.

Energy Decomposition Analysis

Table S11: M06-2X/def2-TZVP Energy Decomposition Analysis and Distortion/Interaction analysis energies of the unconstrained transition states as well as the two constrained versions for the consistent geometry approach, given in kcal/mol.

	**benzene**	**toluene**	**anisole**	**thioanisole**	***N,N*-dimethylaniline**	**Nor/DMT**
				**unconstrained TS**		
$\Delta E_{\mathrm{dist,diene}}$	32.6	32.5	34.8	34.0	26.1	17.9
$\Delta E_{\mathrm{dist,dienophile}}$	18.4	17.9	19.1	18.4	13.9	7.8
ΔE_{dist}	**51.0**	**50.5**	**53.9**	**52.3**	**40.0**	**25.6**
ΔE_{int}	**−27.4**	**−28.7**	**−33.6**	**−31.2**	**−25.6**	**−14.9**
ΔE_{OI}	−101.1	−100.7	−116.1	−109.9	−83.9	−54.9
$\Delta V_{\mathrm{elstat}}$	−189.1	−189.6	−208.3	−198.0	−160.8	−137.2
$\Delta E_{\mathrm{Pauli}}$	262.4	261.3	291.2	276.9	219.1	177.0
$\Delta E^{\ddagger}$	**23.6**	**21.8**	**20.3**	**21.1**	**14.5**	**10.7**
				**synch-TS**		
$\Delta E_{\mathrm{dist,diene}}$	32.6	30.8	36.2	36.1	36.6	37.3
$\Delta E_{\mathrm{dist,dienophile}}$	18.4	16.7	20.2	20.3	19.5	18.0
ΔE_{dist}	**51.0**	**47.5**	**56.4**	**56.4**	**56.1**	**55.3**
ΔE_{int}	**−27.4**	**−25.7**	**−36.4**	**−35.4**	**−36.9**	**−49.3**
ΔE_{OI}	−101.1	−95.1	−111.6	−111.1	−111.8	−115.7
$\Delta V_{\mathrm{elstat}}$	−189.1	−183.8	−200.6	−197.6	−205.9	−203.7
$\Delta E_{\mathrm{Pauli}}$	262.4	252.8	275.5	273.0	280.5	270.2
$\Delta E^{\ddagger}$	**23.6**	**21.7**	**20.1**	**21.0**	**19.2**	**6.0**
				**asynch-TS**		
$\Delta E_{\mathrm{dist,diene}}$	20.3	21.1	23.2	22.9	26.1	19.7
$\Delta E_{\mathrm{dist,dienophile}}$	8.4	8.8	10.7	10.3	13.9	10.0
ΔE_{dist}	**28.7**	**29.9**	**33.9**	**33.2**	**40.0**	**29.6**
ΔE_{int}	**−3.5**	**−7.2**	**−14.4**	**−12.5**	**−25.6**	**−9.0**
ΔE_{OI}	−67.3	−69.9	−75.7	−74.3	−83.9	−66.2
$\Delta V_{\mathrm{elstat}}$	−149.9	−152.3	−155.6	−152.0	−160.8	−153.9
$\Delta E_{\mathrm{Pauli}}$	213.3	214.8	216.8	213.6	219.1	211.0
$\Delta E^{\ddagger}$	**25.2**	**22.7**	**19.6**	**20.7**	**14.5**	**20.6**

Comparison of Different Tetrazines

Table S12: Energies for the Diels–Alder reactions between arenes **1** and **5** and **CF_3Tz**,**$COOCH_3$Tz**, and **PhTz** given in Eh. In CPCM solvent.

		M06-2X			DLPNO-CCSD(T)
		298.15 K	383.15 K	413.15 K	
			**benzene**		
**$SM_{benzene}$**	ΔE		−232.2277308		−231.8052468
	ΔH	−232.1181578	−232.1169129	−232.1169129	
	ΔG	−232.1532352	−232.1627549	−232.1662934	
**SM_{COOCH_3Tz}**	ΔE		−752.0804367		−750.9120833
	ΔH	−751.9211706	−751.9184682	−751.9184682	
	ΔG	−751.9807432	−751.9960471	−752.0018195	
**$TS_{benzene,COOCH_3Tz}$**	ΔE		−984.2671025		−982.6825855
	ΔH	−983.9973903	−983.9933683	−983.9933683	
	ΔG	−984.0687111	−984.0867098	−984.0936147	
**SM_{PhTz}**	ΔE		−758.4239183		−757.0913048
	ΔH	−758.1861846	−758.1827691	−758.1827691	
	ΔG	−758.2487353	−758.2646109	−758.2706783	
**$TS_{benzene,PhTz}$**	ΔE		−758.4239183		−757.0913048
	ΔH	−758.1861846	−758.1827691	−758.1827691	
	ΔG	−758.2487353	−758.2646109	−758.2706783	
			***N,N*-dimethylaniline**		
**$SM_{dimethylaniline}$**	ΔE		−366.1872482		−365.5410613
	ΔH	−365.9977462	−365.9955953	−365.9955953	
	ΔG	−366.0451639	−366.0576498	−366.0623590	
**SM_{COOCH_3Tz}**	ΔE		−752.0855163		−750.9169198
	ΔH	−751.9262865	−751.9235828	−751.9235828	
	ΔG	−751.9859019	−752.0012236	−752.0070029	
**$TS_{dimethylaniline,COOCH_3Tz}$**	ΔE		−1118.250939		−1116.438884
	ΔH	−1117.900874	−1117.895944	−1117.895944	
	ΔG	−1117.983273	−1118.003734	−1118.011625	
**SM_{PhTz}**	ΔE		−758.4264072		−757.0936607
	ΔH	−758.1887701	−758.1853518	−758.1853518	
	ΔG	−758.2518622	−758.2678801	−758.2739977	
**$TS_{dimethylaniline,PhTz}$**	ΔE		−1124.560259		−1122.591613
	ΔH	−1124.134490	−1124.128887	−1124.128887	
	ΔG	−1124.217294	−1124.237858	−1124.245884	

Table S13: M06-2X/def2-TZVP activation energies for benzene as well as *N,N*-dimethylaniline with three different tetrazines, given in kcal/mol. In CPCM solvent.

		$\Delta E^{\ddagger}$	$\Delta H^{\ddagger}$			$\Delta G^{\ddagger}$		
			298.15 K	383.15 K	413.15 K	298.15 K	383.15 K	413.15 K
**benzene**	**CF_3Tz**	23.9	28.3	26	24.6	39.6	43.9	45.5
	**$COOCH_3$Tz**	25.8	26.9	26.9	26.9	41.0	45.2	46.7
	**PhTz**	34.6	35.1	35.1	35.1	50.4	54.9	56.5
***N,N*-dimethylaniline**	**CF_3Tz**	10.7	12.3	12.4	12.4	27.2	31.8	33.5
	**$COOCH_3$Tz**	13.7	14.5	14.6	14.6	30.0	34.6	36.2
	**PhTz**	33.5	32.6	32.7	32.7	50.0	55.0	56.8

Table S14: DLPNO-CCSD(T) corrected activation energies for benzene as well as *N,N*-dimethylaniline with three different tetrazines, given in kcal/mol. In CPCM solvent.

		$\Delta E^{\ddagger}$	$\Delta H^{\ddagger}$			$\Delta G^{\ddagger}$		
			298.15 K	383.15 K	413.15 K	298.15 K	383.15 K	413.15 K
**benzene**	**CF_3Tz**	20.9	25.3	23.1	21.6	36.6	41.0	42.5
	**$COOCH_3$Tz**	21.8	22.4	22.4	22.4	37.0	41.3	42.8
	**PhTz**	29.3	29.8	29.8	29.8	45.1	49.6	51.2
***N,N*-dimethylaniline**	**CF_3Tz**	10.2	11.1	11.1	11.1	26.7	31.3	32.9
	**$COOCH_3$Tz**	12.0	12.8	12.9	12.9	28.3	32.9	34.5
	**PhTz**	27.1	26.2	26.2	26.2	43.6	48.6	50.3
